# Supplementary material for: The Therapeutic Effects of EFNB2-Fc in a Cell Model of Kawasaki Disease
Source: Pharmaceuticals (Basel). 2023 Mar 28;16(4):500. doi: 10.3390/ph16040500 (PMC10142267; doi:10.3390/ph16040500)
Supplement: Supplementary file 1 [file pharmaceuticals-16-00500-s001.zip › pharmaceuticals-2246647-supplementary.pdf]

Supplementary Table S1: Clinical characteristics and laboratory findings in KD patients and healthy controls

Characteristics	Kawasaki disease				Healthy Control
	KD with CAA	*p* value	KD without CAA	*p* value	
Male/female	6/2		11/7		5/15
Age, month	32.63 ± 6.24	0.895	31.44 ± 4.51	0.936	29.67 ± 2.43
Weight (kg)	13.75 ± 1.84	0.577	13.21 ± 0.87	0.708	12.34 ± 0.40
Days of fever	5.13 ± 0.55		5.06 ± 0.32		-
Other symptoms					
Rash	6/8		15/18		-
Conjunctivitis	8/8		17/18		-
Oral changes	7/8		18/18		-
Palmar and plantar erythema	3/8		7/18		-
Cervical adenopathy	7/8		16/18		-
WBC ($\times 10^9$/L)	15.17 ± 2.19	0.002	14.00 ± 1.20	0.001	8.69 ± 0.48
N ($\times 10^9$/L)	11.87 ± 1.35	<0.0001	9.72 ± 1.41	0.0001	3.15 ± 0.27
N (%)	80.13 ± 2.66	<0.0001	64.88 ± 4.75*	<0.0001	35.83 ± 2.10
PLT ($\times 10^9$/L)	340.50 ± 32.33	0.719	364.90 ± 28.46	0.393	309.30 ± 19.63
CRP (mg/L)	75.71 ± 14.33	<0.0001	47.40 ± 7.84	<0.0001	1.05 ± 0.59
ESR (mm/h)	44.38 ± 7.95		60.61 ± 5.46		-
IL-2 (pg/mL)	3.40 (3.18, 4.40)		3.36 (2.78, 3.50)		-
IL-4 (pg/mL)	2.95 (2.60, 3.30)		2.33 (2.03, 2.60) **		-
IL-6 (pg/mL)	231.20 (72.93, 610.90)		65.40 (22.85, 178.10)		-
IL-10 (pg/mL)	18.10 (8.03, 78.73)		7.25 (3.20, 14.31) *		-
TNF-α (pg/mL)	1.25 (1.00, 3.43)		1.50 (1.20, 1.72)		-
IFN-γ (pg/mL)	6.30 (4.93, 21.83)		2.70 (1.70, 2.95) ****		-

All *p* values are for patients compared with healthy controls. $^{*}p<0.05$, $^{**}p<0.01$, $^{****}p<0.0001$ versus KD with CAA. WBC, white blood cell; N, neutrophil; PLT, platelet; CRP, C-reactive protein; ESR, erythrocyte sedimentation rate; IL, interleukin.

Supplementary Figure S1: Full-length blots

Full-length blots for Figure 2

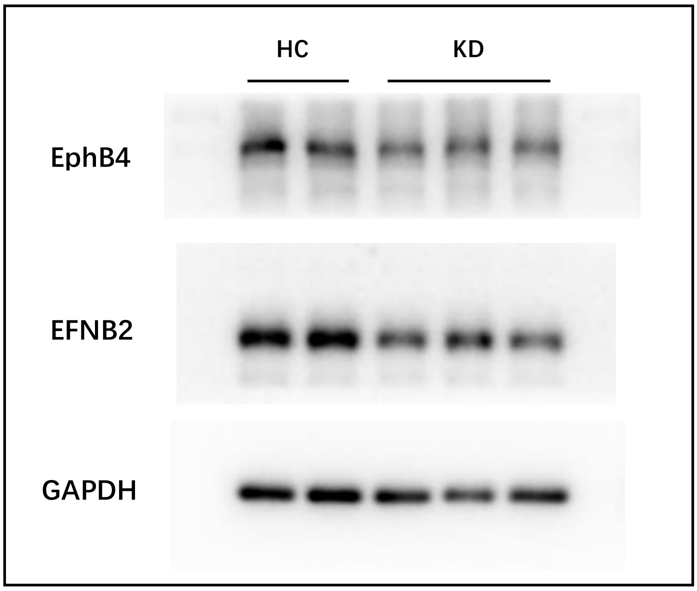

Full-length blots for Figure 3

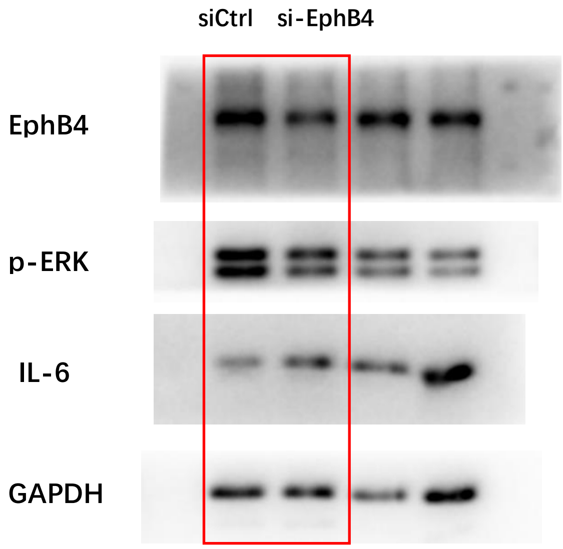

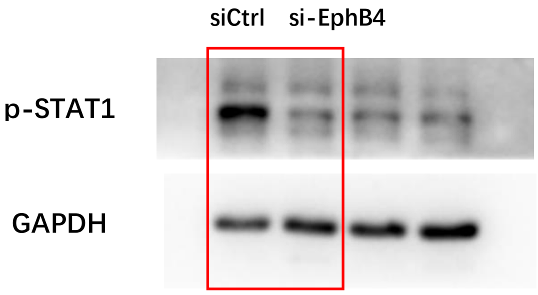

Full-length blots for Figure 4

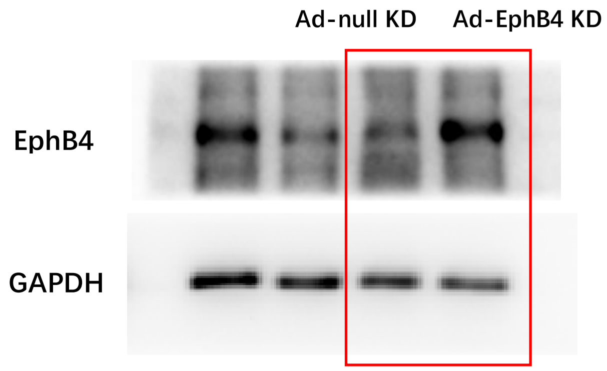

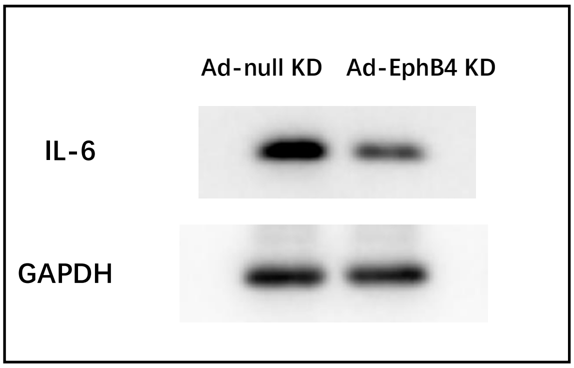

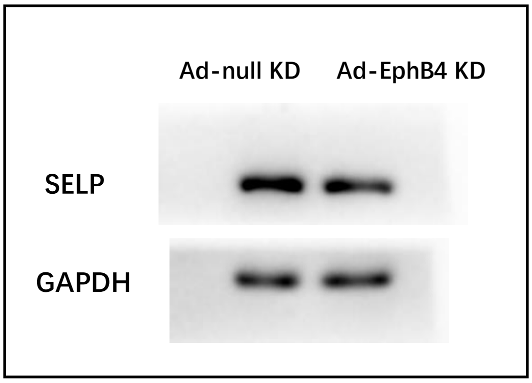

Full-length blots for Figure 5

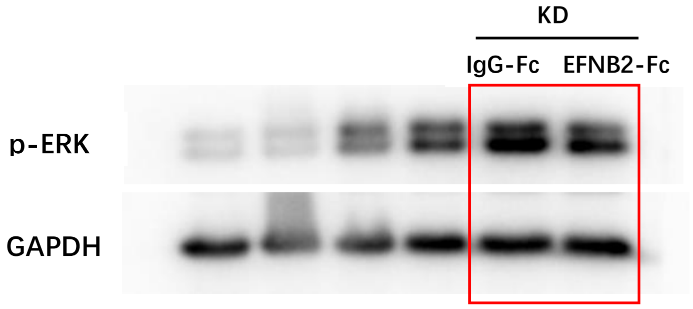

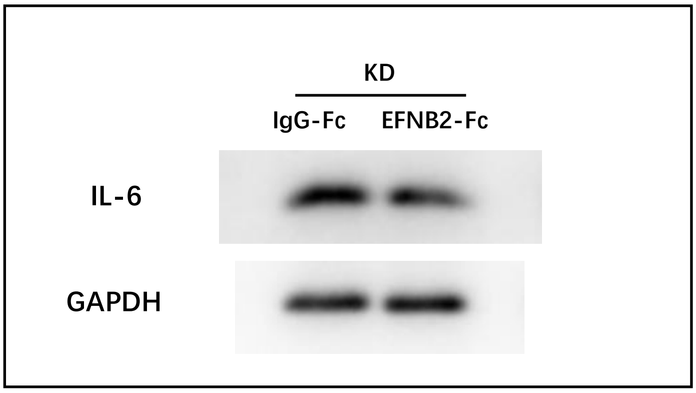
KD
IgG-Fc
EFNB2-Fc
IL-6
GAPDH

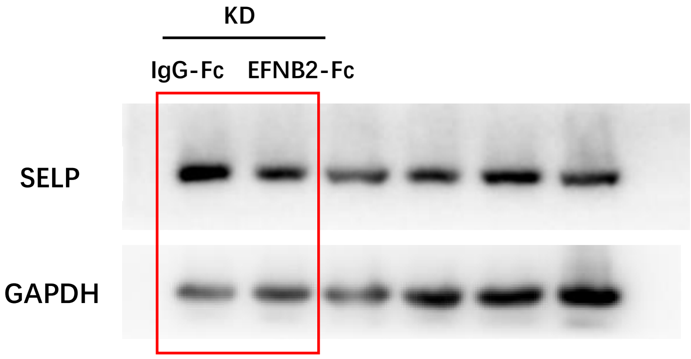
KD
IgG-Fc
EFNB2-Fc
SELP
GAPDH
